# The k conditional nearest neighbor algorithm for classification and class probability estimation

**DOI:** 10.7717/peerj-cs.194

**Published:** 2019-05-13

**Authors:** Hyukjun Gweon, Matthias Schonlau, Stefan H. Steiner

**Affiliations:** 1 University of Western Ontario, London, Canada; 2 University of Waterloo, Waterloo, Canada

**Keywords:** Nonparametric classification, Nearest neighbor, Probabilistic classifier

## Abstract

The k nearest neighbor (kNN) approach is a simple and effective nonparametric algorithm for classification. One of the drawbacks of kNN is that the method can only give coarse estimates of class probabilities, particularly for low values of k. To avoid this drawback, we propose a new nonparametric classification method based on nearest neighbors conditional on each class: the proposed approach calculates the distance between a new instance and the kth nearest neighbor from each class, estimates posterior probabilities of class memberships using the distances, and assigns the instance to the class with the largest posterior. We prove that the proposed approach converges to the Bayes classifier as the size of the training data increases. Further, we extend the proposed approach to an ensemble method. Experiments on benchmark data sets show that both the proposed approach and the ensemble version of the proposed approach on average outperform kNN, weighted kNN, probabilistic kNN and two similar algorithms (LMkNN and MLM-kHNN) in terms of the error rate. A simulation shows that kCNN may be useful for estimating posterior probabilities when the class distributions overlap.

## Introduction

Supervised classification is a fundamental problem in supervised learning. A common approach to classification is to assume a distribution for each different class. Nonparametric classifiers are often used when it is difficult to make assumptions about the class distribution for the problem. The *k*-nearest neighbor (*kNN*) approach (Fix & Hodges, 1951) is one of the most popular nonparametric approaches (Wu et al., 2008). For an input **x**, the *kNN* algorithm identifies *k* objects in the training data that are closest to **x** with a predefined metric and makes a prediction by majority vote from the classes of the *k* objects. Although the *kNN* method is simple and does not require a priori knowledge about the class distributions, *kNN* has been successfully applied in many problems such as character recognition (Belongie, Malik & Puzicha, 2002) and image processing (Mensink et al., 2013). A number of experiments on different classification problems have demonstrated its competitive performance (Ripley, 2007). A detailed survey of the literature about *kNN* can be found in (Bhatia & Vandana, 2010).

One of the drawbacks of *kNN* is that the method can only give coarse estimates of class probabilities particularly for low values of *k*. For example, with two neighbours or *k* = 2 the estimated probabilities can only take the values 0%, 50% or 100% depending on whether 0, 1 or 2 neighbors belong to the class. A probabilistic *kNN* method (*PNN*) was proposed in (Holmes & Adams, 2002) for continuous probability estimates. However, *PNN* and *kNN* are comparable in terms of classification accuracy, and *PNN* has greater computational costs than *kNN* (Manocha & Girolami, 2007).

Many other extensions of *kNN* have been proposed to improve prediction of classification. One direction is to assign different weights to the *k* nearest neighbors based on their distances to the input **x**. Higher weights are given to neighbors with lower distances. Examples include weighted *kNN* (*WkNN*) (Dudani, 1976) and fuzzy *kNN* (Keller, Gray & Givens, 1985). Another approach to improve the prediction of *kNN* is to use the class local means. One of the successful extensions is the local mean based *k* nearest neighbor approach (*LMkNN*) (Mitani & Hamamoto, 2006). For a new test instance **x**, *LMkNN* finds the *k* nearest neighbors in each class and calculates the local mean vector of the *k* nearest neighbors. The distance between **x** and each local mean is calculated and the class corresponding to the smallest distance is assigned to **x**. Empirical evidence suggests that compared to *kNN*, *LMkNN* is robust to outliers when the training data are small (Mitani & Hamamoto, 2006). The idea of *LMkNN* has been applied to many other methods such as pseudo nearest neighbor (Zeng, Yang & Zhao, 2009), group-based classification (Samsudin & Bradley, 2010) and local mean-based pseudo *k*-nearest neighbor (Gou et al., 2014). Recently, an extension of *LMkNN*, the multi-local means-based *k*-harmonic nearest neighbor (*MLM*-*kHNN*) (Pan, Wang & Ku, 2017), was introduced. Unlike *LMkNN*, *MLM*-*kHNN* computes *k* different local mean vectors in each class. *MLM*-*kHNN* calculates their harmonic mean distance to **x** and assigns the class with the minimum distance. An experimental study showed that *MLM*-*kHNN* achieves high classification accuracy and is less sensitive to the parameter *k*, compared to other *kNN*-based methods. However, those local mean based approaches only produce scores for classification and thus are not appropriate when class probabilities are desired.

In this paper, we propose a new nonparametric classifier, *k* conditional nearest neighbor (*kCNN*), based on nearest neighbors conditional on each class. For any positive integer *k*, the proposed method estimates posterior probabilities using only the *k*th nearest neighbor in each class. This approach produces continuous class probability estimates at any value of *k* and thus is advantageous over *kNN* when posterior probability estimations are required. We show that classification based on those posteriors is approximately Bayes optimal for a two-class problem. Furthermore, we demonstrate that the classification approach converges in probability to the Bayes classifier as the size of the training data increases. We also introduce an ensemble of *kCNN* (*EkCNN*) that combines *kCNN* classifiers with different values for *k*. Our experiments on benchmark data sets show that the proposed methods perform, on average, better than *kNN*, *WkNN*, *LMkNN* and *MLM*-*kHNN* in terms of the error rate. Further analysis also shows that the proposed method is especially advantageous when (i) accurate class probabilities are required, and (ii) class distributions overlap. An application using text data shows that the proposed method may outperform *kNN* for semi-automated classification.

The algorithm proposed in this paper is meant for situations in which nearest neighbor-type algorithms are attractive, i.e., for highly nonlinear functions where the training data and the number of features are not too large. Other approaches such as Support Vector Machines (Vapnik, 2000) and Random Forest (Breiman & Schapire, 2001) are therefore not considered.

The rest of this paper is organized as follows: in ‘Methods’, we present the details of the proposed method. In ‘Experimental Evaluation’ , we report on experiments that compare the proposed method with other algorithms using benchmark data sets. In ‘Exploring Properties of the Proposed Method’ simulation, we investigate how the decision boundary and probability field of the proposed method vary using simulation data. In ‘Application: semi-automated classification using the Patient Joe text data’, we apply the proposed method to semi-automated classification using “Patient Joe” text data. In ‘Discussion’, we discuss the results. In ‘Conclusion’, we draw conclusions.

## Methods

### K conditional nearest neighbor

**Figure 1:**
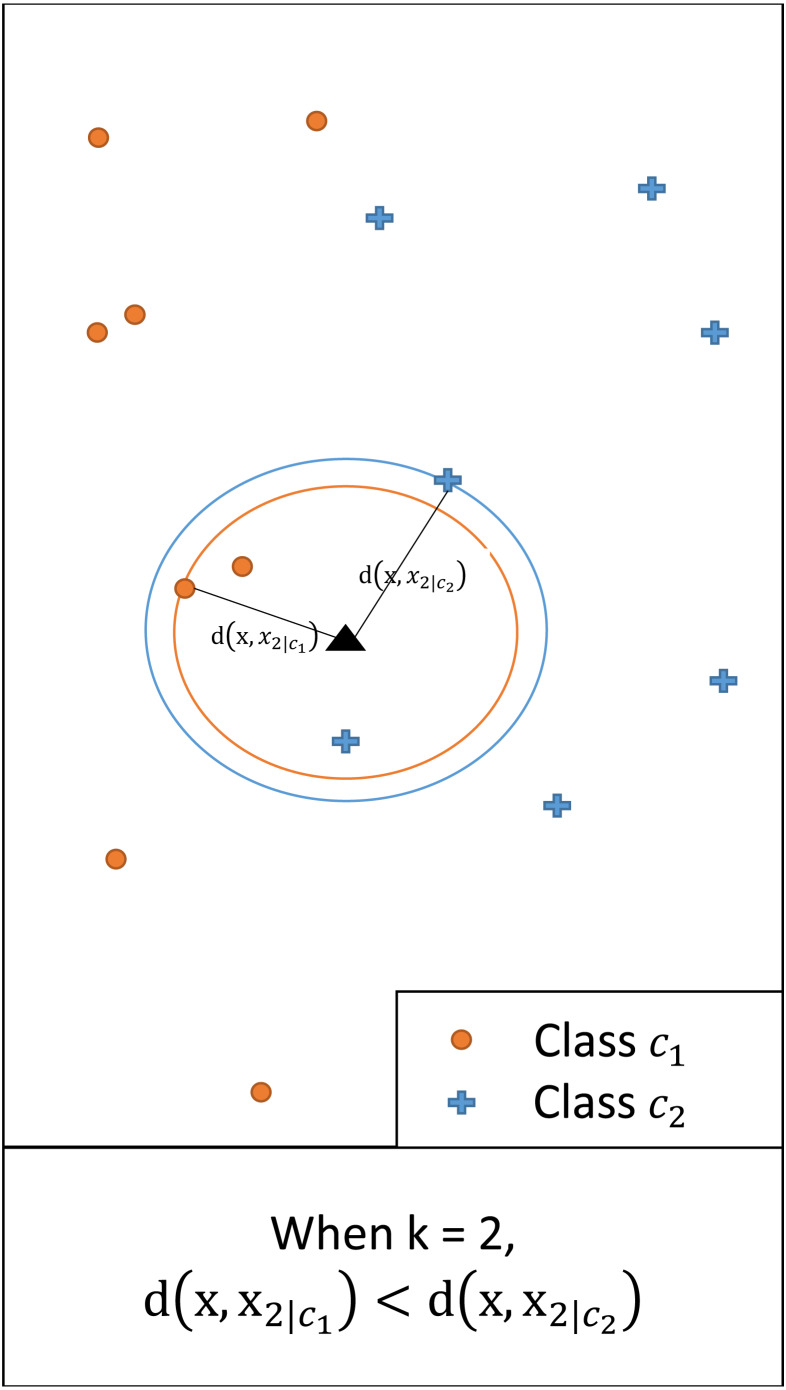
An illustrative example of *d*(**x**, **x**_*k*|*i*_), *i* = 1,2, when *k* = 2. Since the distance for class *c*_1_ is smaller, for the given query class *c*_1_ has a larger posterior probability than *c*_2_.

In multi-class classification, an instance with a feature vector **x** ∈ ℝ^*q*^ is associated with one of the possible classes *c*_1_, …, *c*_*L*_. We assume a set of training data containing *N* classified instances. For any **x** and a given *k*, we denote by **x**_*k*|*i*_ the *k*th nearest neighbor of class *c*_*i*_ (*i* = 1, …, *L*). Let *d*(**x**, **x**_*k*|*i*_) = |**x** − **x**_*k*|*i*_| be the (Euclidean) distance between **x** and **x**_*k*|*i*_. Figure 1 illustrates this showing the distance between **x** and the second nearest neighbor (i.e., *k* = 2) of each class.

Consider a hypersphere with radius *d*(**x**, **x**_*k*|*i*_) centered at **x**. By the definition of **x**_*k*|*i*_, the hypersphere contains *k* instances of class *c*_*i*_. We may approximate the local conditional density *f*(**x**|*c*_*i*_) as

(1) \begin{eqnarray*}\hat {f}(\mathbf{x}{|}{c}_{i})& = \frac{k}{{N}_{i}{V}_{k{|}i}} \end{eqnarray*}

where *V*_*k*|*i*_ is the volume of a hypersphere with radius *d*(**x**, **x**_*k*|*i*_) centered at **x** and *N*_*i*_ represents the number of instances classified as class *c*_*i*_. This approximation was also introduced in Fukunaga & Hostetler (1975). The approximation assumes that *f*(**x**|*c*_*i*_) is nearly constant within the hypersphere of volume *V*_*k*|*i*_ when the radius *d*(**x**, **x**_*k*|*i*_) is small. Using the prior $\hat {p}({c}_{i})\approx \frac{{N}_{i}}{N} $ where $N={\mathop{\sum }\nolimits }_{i=1}^{L}{N}_{i}$ and Bayes theorem, the approximate posterior may be obtained as

(2) \begin{eqnarray*}{\hat {p}}_{k}({c}_{i}{|}\mathbf{x})& = \frac{\hat {p}({c}_{i})\hat {f}(\mathbf{x}{|}{c}_{i})}{\hat {f}(\mathbf{x})} = \frac{1}{\hat {f}(\mathbf{x})} \frac{k}{N{V}_{k{|}i}} .\end{eqnarray*}

Because ${\mathop{\sum }\nolimits }_{i=1}^{L}\hat {p}({c}_{i}{|}\mathbf{x})=1$, we have $\hat {f}(\mathbf{x})={\mathop{\sum }\nolimits }_{i=1}^{L} \frac{k}{N{V}_{k{|}i}} $. Then, ${\hat {p}}_{k}({c}_{i}{|}\mathbf{x})$ may be obtained as

(3) \begin{eqnarray*}{\hat {p}}_{k}({c}_{i}{|}\mathbf{x})& = \frac{ \frac{k}{N{V}_{k{|}i}} }{\sum _{j=1}^{L} \frac{k}{N{V}_{k{|}j}} } = \frac{{d(\mathbf{x},{\mathbf{x}}_{k{|}i})}^{-q}}{\sum _{j=1}^{L}{d(\mathbf{x},{\mathbf{x}}_{k{|}j})}^{-q}} \end{eqnarray*}

since *V*_*k*|*i*_ ∝ *d*(**x**, **x**_*k*|*i*_)^*q*^. The class with the shortest distance among the *L* distances has the highest posterior.

The results in Eq. (3) are affected by the dimension of the feature space (*q*); the class probabilities converge to binary output (1 if the distance is smallest and 0 otherwise) as *q* increases. This implies the estimated class probabilities will be extreme in high-dimensional data, which is not desirable especially when the confidence of a prediction is required. Since smoothing parameters can improve predictive probability accuracy (e.g., LaPlace smoothing for the Naive Bayes algorithm (Mitchell, 1997), we introduce an optional tuning parameter *r* as follows:

(4) \begin{eqnarray*}{\hat {p}}_{k}({c}_{i}{|}\mathbf{x})& = \frac{{d(\mathbf{x},{\mathbf{x}}_{k{|}i})}^{-q/r}}{\sum _{j=1}^{L}{d(\mathbf{x},{\mathbf{x}}_{k{|}j})}^{-q/r}} \end{eqnarray*}

where *r* ≥ 1 controls the influence of the dimension of the feature space *q*. As *r* increases, each posterior converges to 1∕*L*. That is, increasing *r* smoothes the posterior estimates.

The *k* conditional nearest neighbor (*kCNN*) approach classifies **x** into the class with the largest estimated posterior probability. That is, class $\hat {c}$ is assigned to **x** if

\begin{eqnarray*}\hat {c}={argmax}_{i} {\hat {p}}_{k}({c}_{i}{|}\mathbf{x}). \end{eqnarray*}

The proposed classifier is equivalent to *kNN* when *k* = 1. We summarize the *kCNN* classifier in Algorithm 1.

Note that *r* affects the class probabilities but not the classification. We will show in ‘Ensemble of *kCNN*’ that the tuning parameter affects the classification of the ensemble of *kCNN*, which is presented in ‘Ensemble of kCNN’.

 
_________________________________________________________________________________________________ 
 Algorithm 1: The k conditional nearest neighbor algorithm 
_________________________________________________________________________________________________ 
      Input: A training data set D, a new instance vector x with dimension q, a positive inte 
      ger k, parameter r, a distance metric d 
      for i = 1 to L do 
         (a) From D, select xk|i, the kth nearest neighbor of x for class ci 
         (b) Calculate d(x,xk|i), the distance between x and xk|i 
      end for 
      for i = 1 to L do 
        Obtain ˆ pk(ci|x) ←  d(x,xk|i)−q∕r ____∑Lj=1 d(x,xk|j)−q∕r 
      end for 
      Classify x into ˆ c if ˆ c = argmax i ˆ pk(ci|x) 
__________________________________________________________________________________________________    

**Figure 2:**
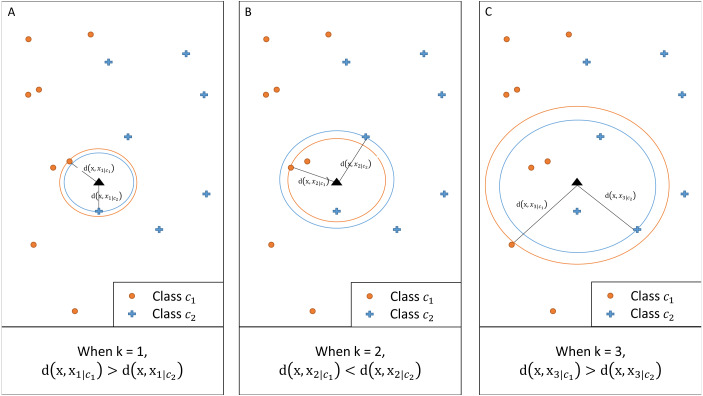
Illustration of *kCNN* at different values of *k*. For any given *k*, class with a shorter distance has a larger class probability.

Figure 2 illustrates an example of a two-class classification problem. For a given *k*, the method calculates the distance between **x** and the *k*th nearest neighbor of each class. When *k* = 1 and *k* = 3, class *c*_2_ has a larger posterior probability than *c*_1_ as the corresponding distance is shorter. When *k* = 2, however, the posterior probability for class *c*_1_ is greater.

### Convergence of *kCNN*

The following theorem says that as the training data increase, *kCNN* converges to the optimal classifier, the Bayes classifier.

**Theorem (convergence of *kCNN*):** Consider a two-class problem with *c*_1_ and *c*_2_ where *p*(*c*_1_) > 0 and *p*(*c*_2_) > 0. Assume that *f*(**x**|*c*_*i*_) (*i* = 1, 2) is continuous on ℝ^*q*^. If the following conditions (a) *k* → ∞, and (b) $ \frac{k}{{\min }_{i}{N}_{i}} \rightarrow 0$ are satisfied, then for any **x** where *f*(**x**) > 0, *kCNN* with *r* = 1 converges in probability to the Bayes classifier.

*Proof:* Since *kCNN* makes predictions by approximate posteriors in Eq. (2), it is sufficient to show that ${\hat {p}}_{k}({c}_{i}{|}\mathbf{x})$ converges in probability to the true posterior.

We first consider the convergence of the prior estimate $\hat {p}({c}_{i})={N}_{i}/N$. Let *c*^(*j*)^ be the class of the *j*th training instance. The prior estimate may be described as $\hat {p}({c}_{i})= \frac{1}{N} {\mathop{\sum }\nolimits }_{j=1}^{N}I({c}^{(j)}={c}_{i})$ where *I* is the indicator function. Hence, by the weak law of large numbers, $\hat {p}({c}_{i})\rightarrow _{}^{p}$ ^1^ 1$A\rightarrow _{}^{p}B$ means *A* converges in probability to *B*.*p*(*c*_*i*_).

We next show that the approximation $\hat {f}(\mathbf{x}{|}{c}_{i})$ in equation Eq. (1) converges in probability to the true conditional density function. Let ${f}_{N}(\mathbf{x})= \frac{k}{NV} $ be an estimate of the density function *f*(**x**) where *V* is the volume of the hypersphere centered at **x** containing *k* training instances. In Loftsgaarden & Quesenberry (1965), it is showed that *f*_*N*_(**x**) converges in probability to *f*(**x**) if *k* → ∞ and $ \frac{k}{N} \rightarrow 0$ as *N* increases. We may apply this result to the convergence of the conditional density functions. By the second condition, both $ \frac{k}{{N}_{1}} $ and $ \frac{k}{{N}_{2}} $ converge to zero. Hence, $\hat {p}(\mathbf{x}{|}{c}_{i})$ converges in probability to the true conditional density function *f*(**x**|*c*_*i*_).

Since $\hat {p}({c}_{i})\rightarrow _{}^{p}p({c}_{i})$ and $\hat {f}(\mathbf{x}{|}{c}_{i})\rightarrow _{}^{p}f(\mathbf{x}{|}{c}_{i})$,

\begin{eqnarray*}\hat {f}(\mathbf{x})=\sum _{i=1}^{2}\hat {p}({c}_{i})\hat {f}(\mathbf{x}{|}{c}_{i})\rightarrow _{}^{p}\sum _{i=1}^{2}p({c}_{i})f(\mathbf{x}{|}{c}_{i})=f(\mathbf{x}). \end{eqnarray*}

Hence, the approximate posterior in Eq. (2) converges in probability to the true posterior. This implies that *kCNN* converges in probability to the Bayes classifier. ■

The theorem implies that a choice of *k* needs to be subject to conditions (a) and (b) as the size of the data increases.

### Time complexity of *kCNN*

The time complexity of *kNN* is *O*(*Nq* + *Nk*) (Zuo, Zhang & Wang, 2008) (*O*(*Nq*) for computing distances and *O*(*Nk*) for finding the *k* nearest neighbors and completing the classification). In the classification stage, *kCNN* (a) calculates the distances between the test instance to all training instances from each class, (b) identifies the *k*th nearest neighbor from each class, and (c) calculates posterior estimates by comparing the *L* distances and assigns the test instance to the class with the highest posterior estimate. Step (a) requires *O*(*N*_1_*q* + ... + *N*_*L*_*q*) = *O*(*Nq*) multiplications. Step (b) requires *O*(*N*_1_*k* + ... + *N*_*L*_*k*) = *O*(*Nk*) comparisons. Step (c) requires *O*(*L*) sum and comparison operations. Therefore, the time complexity for *kCNN* is *O*(*Nq* + *Nk* + *L*). In practice, the *O*(*L*) component is dominated by the other components, since *L* is usually much smaller than *N*. That is, the difference in the complexities between *kNN* and *kCNN* is small.

### Ensemble of *kCNN*

The illustrative example in Fig. 2 shows that the classification is affected by the choice of *k*. Therefore, we propose an ensemble version of *kCNN* that combines the multiple *kCNN* algorithms with different values of *k*. Ensembles are well known as a method for improving predictive performance (Wu et al., 2008; Rokach, 2010). The ensemble of *k* conditional nearest neighbor (*EkCNN*) method makes a prediction based on the averaged posteriors for different values of *k*. These values are now indexed by *w*: *w* = 1, …, *k*. In the ensemble *EkCNN*, *k* represents the number of ensemble members. Suppose that posterior probability ${\hat {p}}_{w}({c}_{i}{|}\mathbf{x})$ is estimated by Eq. (4) for each *w* = 1, …, *k*. For a new instance **x**, the predicted class $\hat {c}$ is determined by

\begin{eqnarray*}\hat {c}={argmax}_{{c}_{i}} \hat {p}({c}_{i}{|}\mathbf{x})={argmax}_{{c}_{i}}  \frac{1}{k} \sum _{w=1}^{k}{\hat {p}}_{w}({c}_{i}{|}\mathbf{x}). \end{eqnarray*}

**Figure 3:**
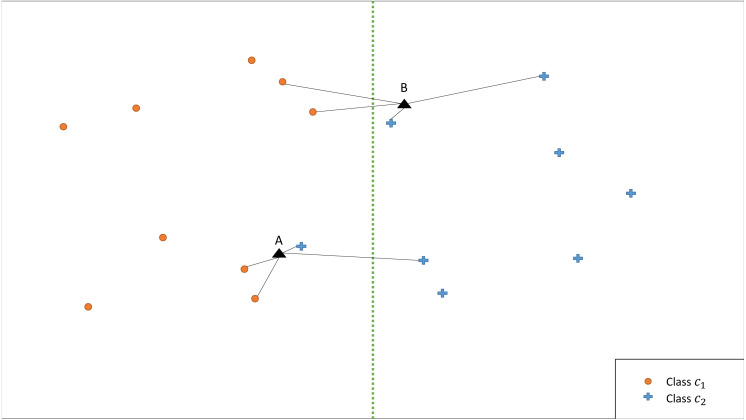
Illustration of classification by *kCNN* versus *EkCNN*. The vertical line is the true class boundary and the target points A and B are to be classified. Based on the distance results, *kCNN* with *k* = 1(*k* = 2) only predicts B (A) correctly. On the other hand, *EkCNN* with *k* = 2 combines class probability for each *k* value and predicts both A and B correctly.

That is, *EkCNN* assigns **x** to the class with the highest average posterior estimate. Unlike *kCNN* that ignores the first *k* − 1 nearest neighbors of each class, *EkCNN* takes into consideration all *k* distances of each class. Using multiple values of *k* makes the prediction less reliant on a single *k*. This may improve the prediction result when the estimated class probabilities are highly variable as a function of *k*. An illustrative example in Fig. 3 shows that *kCNN* predicts either point A or point B incorrectly depending on the choice *k* = 1 or *k* = 2. However, *EkCNN* for *k* = 2 successfully predicts both A and B.

The complexity of *EkCNN* may be obtained analogously to steps (a)–(c) in ‘Time complexity of *kCNN*’. The complexities of *EkCNN* required in step (a) and (b) are the same as those of *kCNN*. In step (c), *EkCNN* requires *O*(*kL*) sum and comparison operations. Hence, the complexity of *EkCNN* is *O*(*Nq* + *Nk* + *kL*).

## Experimental Evaluation

### Data sets

We evaluated the proposed approaches using real benchmark data sets available at the UCI machine learning repository (Lichman, 2013). (We chose data sets to be diverse; all data sets we tried are shown.) Table 1 shows basic statistics of each data set including its numbers of classes and features. All data sets are available online at: https://archive.ics.uci.edu/ml/datasets.html. The data sets are ordered by the number of instances.

**Table 1:** Twenty benchmark data sets and their associated characteristics.

Name	Features	Classes	Instances	Class distributions
Voice	309	2	126	84/42
Wine	13	3	178	71/59/48
Parkins	22	2	195	147/48
Cancer	24	2	198	151/47
Sonar	60	2	208	111/97
Seeds	7	3	210	70/70/70
Haberman	3	2	306	225/81
Ecoli	7	8	336	143/77/52/35/20/5/2/2
Libras	90	15	360	24/24/.../24/24
Musk	166	2	476	269/207
Blood	4	2	748	570/178
Diabetes	8	2	768	500/268
Vehicle	18	4	846	218/217/212/199
German	24	2	1,000	700/300
Yeast	8	10	1,484	463/429/224/163/51/44/35/30/20/5
Handwritten	256	10	1,593	1,441/152
Madelon	500	2	2,000	1,000/1,000
Image	19	7	2,310	330/330/.../330/330
Wave	21	2	5,000	1,696/1,657/1,647
Magic	10	2	19,020	12,332/6,688

### Experimental setup

We compared *kCNN* and *EkCNN* against *kNN*, *WkNN*, *PNN*, *LMkNN* and *MLM*-*kHNN*. Moreover, we considered an ensemble version of *kNN* (*EkNN*). *EkNN* estimates the probability for class *c*_*i*_ as

\begin{eqnarray*}\hat {p}({c}_{i}{|}\mathbf{x})= \frac{1}{k} \sum _{w=1}^{k}{\hat {p}}_{w}({c}_{i}{|}\mathbf{x}) \end{eqnarray*}

where ${\hat {p}}_{w}({c}_{i}{|}\mathbf{x})$ is the probability estimated by *kNN* based on *w* nearest neighbors. Since *MLM*- *kHNN* is an ensemble of *LMkNN* using the harmonic mean, no additional ensemble model was considered. For *EkCNN*, we used *r* = *q* where *q* is the number of features of the data set. For *kCNN* and *EkCNN*, we added *ϵ* = 10^−7^ to each distance in equation Eq. (4) to avoid dividing by zero when the distance is zero.

For evaluation, we choose error rate (or equivalently accuracy), since error rate is one of the most commonly used metric and the skewness of the class distribution is not severe for most of the chosen data sets. The percentage of the majority class is less than 80% for most data sets (19 out of 20 data sets).

The analysis was conducted in *R* (R Core Team, 2014). For assessing the performance of the classifiers, we used 10-fold cross validation for each data. In the experiments, we varied the size of the neighborhood *k* from 1 to 15. For each method except *PNN*, the optimal value of *k* has to be determined based on the training data only. To that end, each training fold of the cross-validation (i.e., 90% of the data) was split into two random parts: internal training data (2/3) and internal validation data (1/3). The optimal *k* was the value that minimized classification error on the internal validation set. For *PNN*, Markov Chain Monte Carlo (*MCMC*) simulated samples are required. Following Holmes & Adams (2002), we used 5,000 burn-in samples, and retained every 100th sample in the next 50,000 samples.

We applied the Wilcoxon signed-rank test (Wilcoxon, 1945; Demšar, 2006) to carry out the pairwise comparisons of the methods over multiple data sets because unlike the t–test it does not make a distributional assumption. Also, the Wilcoxon test is more robust to outliers than the *t*-test (Demšar, 2006). The Wilcoxon test results report whether or not any two methods were ranked differently across data sets. Each test was one-sided at a significance level of 0.05.

### Results

Table 2 summarizes the error rate (or misclassification rate) of each approach on each data set. Parameter *k* was tuned separately for each approach. *EkCNN* performed best on 8 out of the 20 data sets and *kCNN* performed best on 2 data sets. *EkCNN* achieved the lowest (i.e., best) average rank and *kCNN* the second lowest average rank. In the cases where *kCNN* performed the best, *EkCNN* was the second best method. According to the Wilcoxon test, *EkCNN* had a significantly lower (i.e., better) rank than *kNN*, *EkNN*, *WkNN*, *LMkNN* and *kCNN* with *p*-values less than 0.01. There was marginal evidence that *EkCNN* had a lower average rank than *MLM*- *kHNN* (*p*-value = 0.0656). Also, *kCNN* performed significantly better than *kNN* (*p*-value = 0.001), *EkNN* (*p*-value = 0.003), *WkNN* (*p*-value = 0.024), *PNN* (*p*-value = 0.003) and *LMkNN* (*p*-value = 0.041).

**Table 2:** The lowest error rates of each method on benchmark data. “Ranking” refers to the average ranking score of each method over the twenty data sets. Lower is better. Values in bold indicate the best performance in each row.

	*kNN*	*EkNN*	*WkNN*	*PNN*	*LMkNN*	*MLM*- *kHNN*	*kCNN*	*EkCNN*
Voice	**0.3598**	0.3625	0.3990	0.3701	0.4060	0.4316	0.3675	0.3941
Wine	0.2871	0.2748	0.2871	0.3110	0.2819	**0.2361**	0.2770	0.2534
Parkins	0.1783	**0.1583**	0.1750	0.1921	0.1983	0.1833	0.1783	0.1710
Cancer	0.2782	0.3130	0.2942	0.2675	0.3006	0.2927	0.2524	**0.2410**
Sonar	0.1815	0.1815	0.1815	0.2443	0.1820	**0.1534**	0.1767	0.1666
Seeds	0.1500	0.1500	0.1500	0.1423	0.0952	0.1000	0.1000	**0.0901**
Haberman	0.2769	0.2952	0.3128	0.2740	0.3305	0.3388	**0.2572**	0.2604
Ecoli	0.1365	0.1320	0.1370	0.1442	0.1482	0.1335	0.1394	**0.1305**
Libras	0.1528	0.1528	0.1428	0.1405	0.1500	**0.1320**	0.1360	0.1320
Musk	0.1493	0.1444	0.1182	0.1440	**0.0832**	0.0849	0.1388	0.1078
Blood	0.2438	0.2456	0.2397	0.2407	0.2433	0.3208	0.2432	**0.2207**
Diabetes	0.2643	0.2798	0.2736	0.2605	0.2629	0.2759	0.2616	**0.2560**
Vehicle	0.3666	0.3373	0.3721	0.3718	**0.3028**	0.3087	0.3643	0.3560
German	0.3200	0.3220	0.3312	0.3150	0.3200	0.3120	**0.3020**	0.3100
Yeast	0.4192	0.4291	0.4021	0.4024	0.4219	0.4200	0.4152	**0.3943**
Handwritten	0.0891	0.0752	0.0744	0.0901	0.0478	**0.0415**	0.0881	0.0625
Madelon	0.2733	0.2905	0.2939	0.2700	0.3209	0.3592	0.2625	**0.2601**
Image	0.0346	0.0366	0.0330	0.0524	0.0337	**0.0316**	0.0346	0.0346
Wave	0.1590	0.1664	0.1674	**0.1320**	0.1522	0.1606	0.1478	0.1520
Magic	0.1856	0.1833	0.1826	0.1890	0.1962	0.1859	0.1854	**0.1780**
Average	0.2253	0.2265	0.2284	0.2277	0.2239	0.2251	0.2164	**0.2085**
Ranking	5.25	5.38	5.08	5.05	5.22	4.35	3.55	**2.13**

Equation (4) contains a tuning parameter *r*. As mentioned above, increasing *r* smoothes posterior estimates. For the results of *EkCNN* presented in Table 2, we chose *r* = *q* for all data sets. While not shown here, using *r* = *q* resulted in lower or equal error rates compared with using *r* = 1 on 18 out of 20 data sets. Specifying *r* = *q* reduced the error rate up to 6% relative to the error rate for *r* = 1.

### Illustrating the choice of *r* on the sonar data set

**Figure 4:**
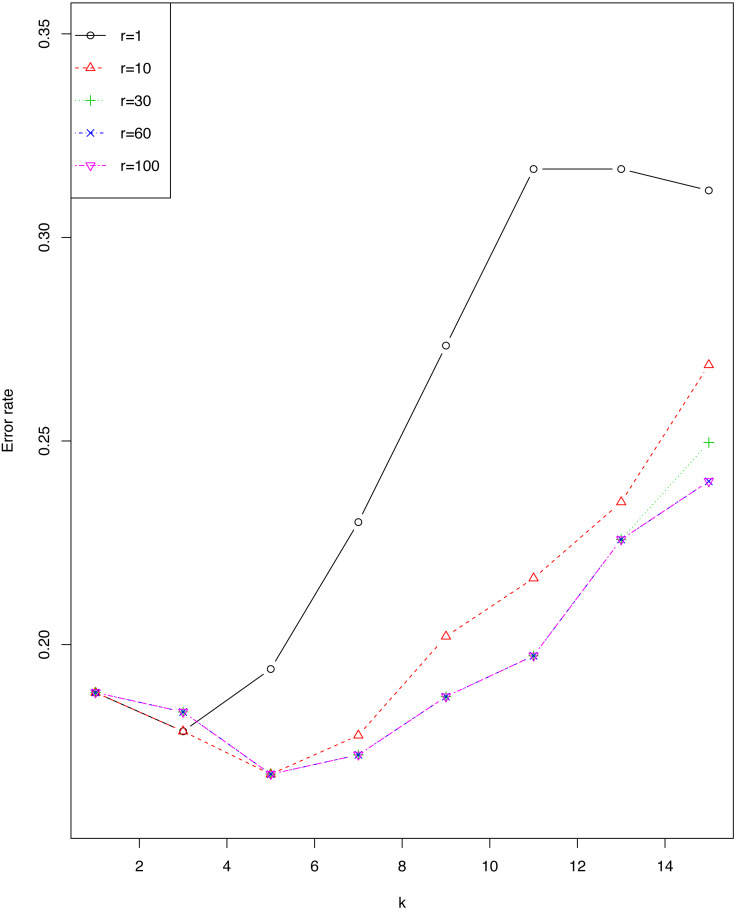
Impact of the tuning parameter *r* on error rates using the *sonar* data set.

We investigated the impact of *r* and *ϵ* on error rate of *EkCNN* (Classification by *kCNN* is affected by neither *r* nor *ϵ*.) for the *sonar* data set. Figure 4 shows that the error rate varied little for small values of *k*. For this data set, larger values of *r* are consistently preferable to smaller values. Note that error rates for *r* = 60 were almost identical to those for *r* = 100.

## Exploring Properties of the Proposed Method

In the following subsections, we investigate *kCNN*’s decision boundary and posterior probability using simulation. Further, we also discuss where *kCNN* beats *kNN* for posterior estimation.

### Decision boundary of *kCNN* and *EkCNN* with varying *k*

This section illustrates that the decision boundary between classes is smoother as *k* increases for both *kCNN* and *EkCNN*. We used a simulated data set from Friedman, Hastie & Tibshirani (2001). The classification problem contains two classes and two real valued features.

**Figure 5:**
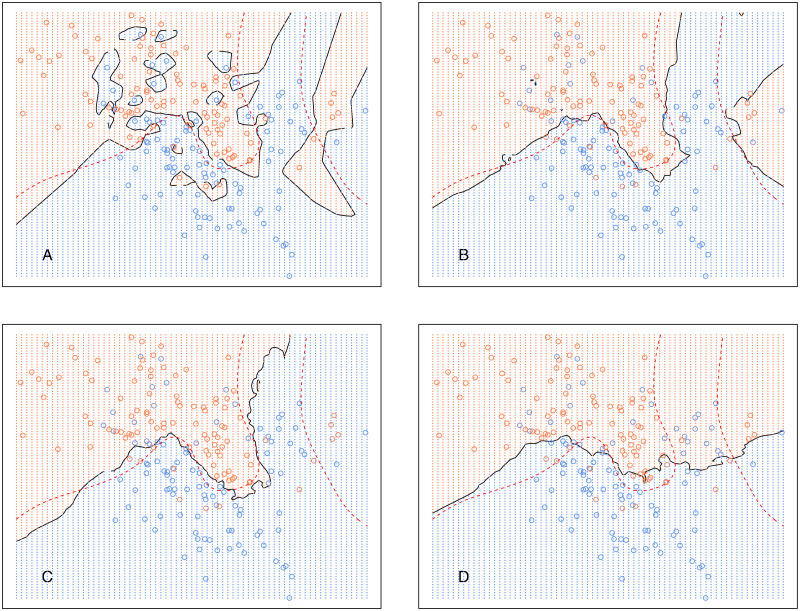
*kCNN* on the simulated data with different choices of *k*. The broken red curve is the Bayes decision boundary. (A) *kCNN* (*k* = 1), (B) *kCNN* (*k* = 5), (C) *kCNN* (*k* = 10), (D) *kCNN* (*k* = 30).

Figure 5 shows the decision boundary of *kCNN* with different *k* (solid curve) and the optimal Bayes decision boundary (dashed red curve). Increasing *k* resulted in smoother decision boundaries. However, when *k* is too large (e.g., *k* = 30 in this example), the decision boundary was overly smooth.

**Figure 6:**
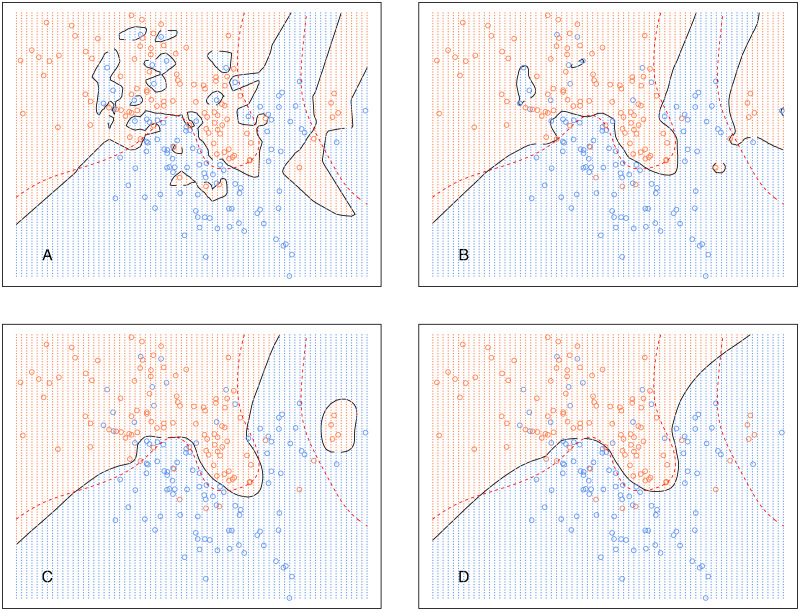
*EkCNN* on the simulated data with different choices of *k*. The broken red curve is the Bayes decision boundary. (A) *EkCNN* (*k* = 1), (B) *EkCNN* (*k* = 5), (C) *EkCNN* (*k* = 10), (D) *EkCNN* (*k* = 30).

Analogously, Fig. 6 shows the decision boundary of *EkCNN* at *r* = 2 and different values *k*. Similar to *kCNN*, the decision boundary was smoothed as *k* increased. However, the magnitude of the changes was less variable. For example, the decision boundaries of *EkCNN* at *k* = 10 and *k* = 30 were similar, while those of *kCNN* were quite different.

### Comparison of the posterior probability distribution of *kNN* and *kCNN*

**Figure 7:**
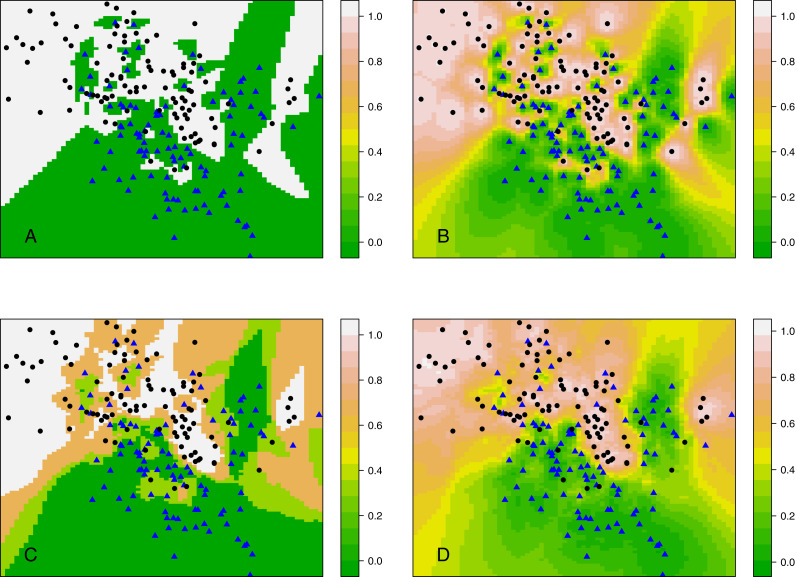
Contour plots of posterior probabilities of *kNN* and *kCNN* for *k* = 1 and *k* = 3. (A) *kNN* (*k* = 1), (B) *kCNN* (*k* = 1), (C) *kNN* (*k* = 3), (D) *kCNN* (*k* = 3).

Rather than considering classification, this section compares *kCNN* with *kNN* in terms of posterior probabilities. Probabilities are of interest, for example, when evaluating the entropy criterion. Using the same data set as in ‘Decision boundary of *kCNN* and *EkCNN* with varying k’, we plot the full posterior probability contours of *kNN* and *kCNN* in Fig. 7. We set *r* = *q* = 2 for *kCNN*. For *k* = 1, as expected, the posteriors estimated by *kNN* was always either 0 or 1. By contrast, *kCNN* provided less extreme posterior results even at *k* = 1. The posterior probabilities changed more gradually.

When *k* = 3, posterior probabilities from *kNN* jumped between four possible values (0, 1/3, 2/3, 1), whereas those from *kCNN* were much smoother. The result shows that unlike *kNN*, *kCNN* can produce smooth posterior probability fields even at small values of *k*.

### Under what circumstances does the proposed method beat *kNN*?

*kCNN* (or *EkCNN*) may be useful when the true posterior distribution has a full range of probabilities rather than near dichotomous probabilities (close to 0 or 1). This occurs when the distributions of the classes substantially overlap. When the distribution of each class is well separated, for any data point the classification probabilities will be (near) 1 for one class and (near) 0 for the other classes. Otherwise, when the distributions overlap, the classification probabilities will be less extreme.

We conducted a small simulation to illustrate that *kCNN* is preferable to *kNN* when *k* is small and the distributions of the classes overlap. Assume that instances from each class are independently distributed following a multivariate normal distribution. Denote by *μ*_*i*_ the mean vector and by ∑_*i*_ the covariance matrix of class *c*_*i*_. The parameters were given as

\begin{eqnarray*}{\mu }_{1}& =(0,0,\ldots ,0), {\sum }_{1}={I}_{q} \end{eqnarray*}

\begin{eqnarray*}{\mu }_{2}& =( \frac{s}{\sqrt{q}} ,\ldots , \frac{s}{\sqrt{q}} ), {\sum }_{2}={I}_{q} \end{eqnarray*}

where *I*_*q*_ is the *q* dimensional identity matrix. Note that *s* is the Euclidean distance between the two means. Therefore, *s* controls the degree of overlap between the distributions of the two classes.

In order to obtain less variable results, we used 10 independent replicates for each parameter setting. The final outputs were obtained by averaging the results. We used 100 training and 1,000 test instances and the equal prior setting for the classes. Like Wu, Lin & Weng (2004), we evaluated the posterior estimates based on mean squared error (*MSE*). The *MSE* for the test data is obtained as

\begin{eqnarray*}MSE= \frac{1}{1000} \frac{1}{2} \sum _{j=1}^{1000}\sum _{i=1}^{2}{ \left( \hat {p}({c}_{i}{|}{\mathbf{x}}_{j})-p({c}_{i}{|}{\mathbf{x}}_{j}) \right) }^{2} \end{eqnarray*}

where **x**_*j*_ represents the *j*th test instance.

Table 3 shows the *MSE* for each method as a function of *s* and *k* when *p* = 2. The *kCNN* method beat *kNN* for small values of *s*. Small values of *s* imply that the mean vectors are close to each other, and hence there is more overlap between the two conditional densities. The difference in performance between the two methods decreased as *s* or *k* increased.

**Table 3:** *MSE* as a function of *k* and *s* for *kNN* and *kCNN*. 100 training instances and *p* = 2 were used. The results were the averages of 10 replicates. Values in bold indicate the best performance in each row.

	*k* = 1		*k* = 5		*k* = 10		*k* = 20	
*s*	*kNN*	*kCNN*	*kNN*	*kCNN*	*kNN*	*kCNN*	*kNN*	*kCNN*
0.1	0.504	**0.074**	0.115	**0.017**	0.065	**0.011**	0.038	**0.006**
0.5	0.483	**0.080**	0.094	**0.022**	0.046	**0.019**	0.025	**0.016**
1	0.449	**0.113**	0.082	**0.054**	**0.042**	0.053	**0.028**	0.058
1.5	0.308	**0.104**	**0.056**	0.064	**0.024**	0.073	**0.016**	0.085
2	0.211	**0.096**	**0.045**	0.082	**0.024**	0.094	**0.016**	0.113

Next, we considered the effect of feature dimension *q* on each method. Table 4 shows the *MSE* for each method as a function of *q* and *k* when *s* = 0.1. Throughout the range of *q*, *kCNN* outperformed *kNN*. As *q* increased the *MSE* for *kCNN* was less affected by the choice of *k*.

**Table 4:** *MSE* as a function of *k* and *q* for *kNN* and *kCNN*. 100 training instances and *s* = 0.1 were used. The results were the averages of 10 replicates. Values in bold indicate the best performance in each row.

	*k* = 1		*k* = 5		*k* = 10		*k* = 20	
*q*	*kNN*	*kCNN*	*kNN*	*kCNN*	*kNN*	*kCNN*	*kNN*	*kCNN*
2	0.502	**0.070**	0.122	**0.014**	0.054	**0.006**	0.022	**0.004**
5	0.499	**0.017**	0.100	**0.003**	0.048	**0.002**	0.021	**0.002**
10	0.503	**0.007**	0.112	**0.003**	0.058	**0.002**	0.027	**0.002**
30	0.500	**0.002**	0.102	**0.002**	0.053	**0.001**	0.026	**0.001**
50	0.494	**0.002**	0.103	**0.001**	0.049	**0.001**	0.023	**0.001**

## Application: Semi-automated Classification Using the Patient Joe Text Data

In the previous section, we discussed situations where the proposed method is preferred over *kNN*. This section shows that the proposed algorithm is useful in the semi-automatic classification of text data. In semi-automatic text classification, high prediction accuracy is more important than fully automating classification; since somewhat uncertain predictions are manually classified. We first distinguish between easy-to-categorize and hard-to-categorize text instances. The easy-to-categorize texts are classified by statistical learning approaches, while the hard-to-categorize instances are classified manually. This is needed especially for text data from open-ended questions in the social sciences, since it is difficult to achieve high overall accuracy with full automation and manual classification is time-consuming and expensive.

The goal in semi-automatic classification is to obtain high classification accuracy for a large number of text instances. Hence, a classifier needs to not only predict the correct classes but also well order the text instances by the difficulty of classification.

For our application, we used a survey text data set (Martin et al., 2011) (we call the data set “Patient Joe”). The data were collected as follows. The respondents were asked to answer the following open-ended question: “Joe’s doctor told him that he would need to return in two weeks to find out whether or not his condition had improved. But when Joe asked the receptionist for an appointment, he was told that it would be over a month before the next available appointment. What should Joe do?” In 2012, the Internet panel LISS (http://www.lissdata.nl) asked the question in Dutch and classified the text answers into four different classes (proactive, somewhat proactive, passive and counterproductive). See Martin et al. (2011) and Schonlau & Couper (2016) for more information about the data set.

The original texts were converted to sets of numerical variables (preprocessing). Briefly, we created an indicator variable for each word (unigram). The variable indicates whether or not the word is present in a text answer. Then a text answer was represented by a binary vector (each dimension represents a word). After converting the text answers in the Patient Joe data set, we had 1758 instances with 1,750 total unigrams.

In semi-automated classification, test instances are ordered from the easiest-to-categorize instance to the hardest-to-categorize instance based on the probability estimate of the predicted class.

**Figure 8:**
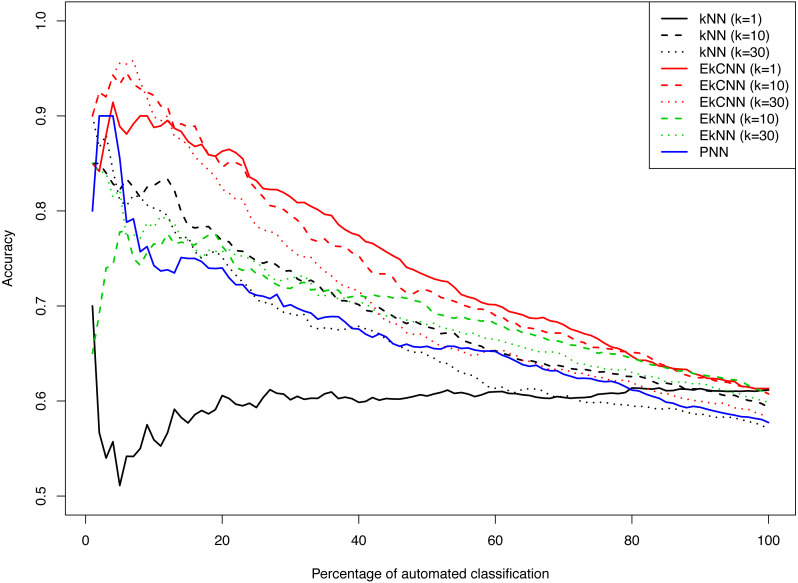
Comparison of *kNN*, *EkNN*, *PNN* and *EkCNN* with different choices of *k* for semi-automatic classification on the Patient Joe data.

Figure 8 shows the accuracy of *kNN*, *EkNN* and *EkCNN* (at *k* = 1, 10 and 30) and *PNN* as a function of the percentage of the test data that were classified automatically by each method. (The other nearest neighbor based approaches, *LMkNN* and *MLM*-*kHNN*, do not produce class probabilities, and thus were excluded in the comparison.) Also, since *EkNN* and *kNN* is equivalent at *k* = 1, the column for *EkNN* with *k* = 1 is omitted. In most cases, high accuracy was achieved when only a small percentage of text answers were classified. However, as the percentage of automated classification increased and more hard-to-categorize instances are included, accuracy tended to decrease. There was one exception: for *kNN* with *k* = 1, accuracy did not increase as the probability threshold for automatic classification increased. That is because for *kNN* at *k* = 1 probability 1 is assigned to the class of the nearest neighbor for each test instance. In other words, for *k* = 1 *kNN* failed to prioritize the text answers. The *EkCNN* method, however, ordered the test instances well even at *k* = 1.*EkCNN* with *k* = 1 resulted in higher accuracy than *k* = 10 or *k* = 30 when more than 20% of the data were classified automatically. From the figure it is clear that *EkCNN* achieved higher accuracy than *kNN* at almost all percentages regardless of the values of *k*. Equivalently, at a target accuracy, a larger number of the text answers could be classified by *EkCNN*. Also *EkCNN* at any value of *k* outperformed *PNN*. The differences in accuracy between the methods tended to be larger at lower percentages of automated classification, i.e., when a substantial percentage of text was manually classified, which is typical in semi-automated classification of open-ended questions. In semi-automated classification this would lead to cost savings. The results are summarized in Table 5. *EkCNN* was preferred to *kNN*, *EkNN* and *PNN* for semi-automated classification of the Patient Joe data.

**Table 5:** Summary statistics for semi-automatic classification for the Patient Joe data. All numbers were the averages of 10 cross validation results. Values in bold indicate the best performance in each row.

Percentage of Automated Classification	Accuracy								
	*kNN*			*EkNN*		*EkCNN*			*PNN*
	*k* = 1	*k* = 10	*k* = 30	*k* = 10	*k* = 30	*k* = 1	*k* = 10	*k* = 30	
10%	0.5591	0.8267	0.80291	0.7653	0.7826	0.8909	**0.9215**	0.8993	0.7424
20%	0.6057	0.7685	0.7514	0.7628	0.7571	**0.8628**	0.8457	0.8228	0.7400
30%	0.6013	0.7371	0.69172	0.7184	0.7296	**0.8147**	0.7957	0.7598	0.7013
40%	0.6005	0.7014	0.6785	0.7114	0.7042	**0.7742**	0.7528	0.7157	0.6757
50%	0.6052	0.6779	0.6484	0.6996	0.6803	**0.7315**	0.7166	0.6666	0.6575
100%	0.6115	0.5932	0.5710	0.6086	0.5990	**0.6132**	0.6074	0.5841	0.5934

## Discussion

For the 20 benchmark data sets, *EkCNN* had the lowest and *kCNN* the second lowest average error rate. In terms of statistical significance, *EkCNN* performed significantly better than *kNN*, *EkNN*, *WkNN*, *PNN*, *LMkNN* and *kCNN* on error rate. For the same data sets, *kCNN* performed significantly better than *kNN*, *EkNN*, *WkNN*, *PNN* and *LMkNN*.

The ensemble method *EkCNN* performed better than *kCNN*. For each *k*, *kCNN* uses a single posterior estimate for each class, whereas *EkCNN* combines multiple posterior estimates. This more differentiated estimate for posteriors may be the reason for the greater classification accuracy. We therefore recommend *EkCNN* over *kCNN* for higher classification accuracy.

We have shown that *kCNN* is asymptotically Bayes optimal for *r* = 1. It is interesting that for the ensemble version *EkCNN*, *r* = *q* is clearly preferable. While surprising, there is no contradiction: the Bayes optimality only applies asymptotically and only for *kCNN* and not for the ensemble version *EkCNN*.

While the tuning parameter *r* does not affect classification for *kCNN*, *r* does affect classification for *EkCNN*. For the empirical results presented in Table 2, we chose *r* = *q* for all data sets. We also noted that in 18 of the 20 data sets *r* = *q* leads to a lower or equal error rate as compared to *r* = 1. Rather than just tuning the parameter *k*, it would be possible to simultaneously tune *k* and *r*. While this may further improve the error rates of *EkCNN*, the improvement, if any, would come at additional computational cost and is not expected to be appreciably large. For example, for the *sonar* data set, we have demonstrated in ‘Illustrating the choice of *r* on the sonar data set’ that no improvement was obtained when *r* > *q*.

The simulation study in ‘Decision boundary of *kCNN* and *EkCNN* with varying *k*’ showed that the decision boundary obtained by *kCNN* can be smoothed by increasing *k*. Although this result seems similar to that of *kNN*, the reasons for smoothed decision boundaries are different. As *k* increases, *kNN* considers more observations for classification and thus the classification is less affected by noise or outliers. By contrast, *kCNN* always uses the same number of observations (the number of classes) to make a prediction regardless of *k*. The *kCNN* approach ignores the first *k* − 1 nearest neighbors from each class and this makes the decision boundary less local.

Since *EkCNN* is a combination of multiple *kCNN* classifiers, its decision boundary is also a combined result of multiple decision boundaries from *kCNN*. Because the decision boundary obtained by *kCNN* is smoothed as *k* increases, that obtained by *EkCNN* is also smoothed. However, the smoothing occurs more gradually, since the decision boundary obtained at *k* is always combined with the *k* − 1 less smooth decision boundaries. This implies that *EkCNN* is more robust than *kCNN* against possible underfitting that may occur at large *k*. The decision boundaries shown in ‘Decision boundary of *kCNN* and *EkCNN* with varying *k*’ confirmed this.

An advantage of the proposed methods over *kNN*, especially when *k* is low, is that *kCNN* (or *EkCNN*) can estimate more fine-grained probability scores than *kNN*, even at low values of *k*. For *kNN*, a class probability for a new observation is estimated as the fraction of observations classified as that class. By contrast, *kCNN* estimates the posteriors based on distances. We confirmed this in ‘Comparison of the posterior probability distribution of *kNN* and *kCNN*’ using simulated probability contour plots.

A simulation in ‘Under what circumstances does the proposed method beat kNN?’ suggests that the greater the overlap among the posterior distribution of each class, the more likely that *kCNN* beats *kNN* in terms of the *MSE*. In most applications class distributions overlap, which partially explains why in the experiment in ‘Result’ *kCNN* performed better than *kNN* in many cases.

The application in ‘Application: Semi-automated Classification Using the Patient Joe Text Data’ showed that *EkCNN* outperformed *kNN* and *PNN* in semi-automated classification, where easy-to-categorize and hard-to-categorize instances need to be separated. When only a percentage of the text data was classified automatically (as is typical in semi-automatic classification), *EkCNN* achieved higher accuracy than the other two approaches.

Like all nearest neighbor approaches, limitations of *kCNN* include lack of scalability to very large data sets.

## Conclusion

In this paper, we have proposed a new nonparametric classification method, *kCNN*, using conditional nearest neighbors. We have demonstrated that *kCNN* is an approximation of the Bayes classifier. Moreover, we have shown that *kCNN* converges in probability to the Bayes optimal classifier as the number of training instances increase. We also considered an ensemble of *kCNN* called *EkCNN*. The proposed methods compared favorably to other nearest neighbor based methods on some benchmark data sets. While not beating all competitors on all data sets, the proposed classifiers are promising algorithms when facing a new prediction task. Also, the proposed methods are especially advantageous when class probability estimations are needed and when the class distributions highly overlap. The proposed method appears especially useful for semi-automated classification.

## Additional Information and Declarations

### Data Availability

The following information was supplied regarding data availability: The R codes have been uploaded at GitHub: https://github.com/hgweon/kcnn. All data used in the manuscript are available at the UCI data repository: https://archive.ics.uci.edu/ml/datasets.php.
